# A multi-species benchmark for training and validating mass spectrometry proteomics machine learning models

**DOI:** 10.1038/s41597-024-04068-4

**Published:** 2024-11-08

**Authors:** Bo Wen, William Stafford Noble

**Affiliations:** 1 Department of Genome Sciences, University of Washington, Seattle, WA USA; 2 Paul G. Allen School of Computer Science and Engineering, University of Washington, Seattle, WA USA

**Keywords:** Machine learning, Proteomics

## Abstract

Training machine learning models for tasks such as *de novo* sequencing or spectral clustering requires large collections of confidently identified spectra. Here we describe a dataset of 2.8 million high-confidence peptide-spectrum matches derived from nine different species. The dataset is based on a previously described benchmark but has been re-processed to ensure consistent data quality and enforce separation of training and test peptides.

## Background & Summary

*De novo* sequencing of proteomics tandem mass spectrometry data, in which observed fragmentation spectra are translated into corresponding peptide sequences, has been an open challenge for more than 40 years^1^. Recently, as in many other areas of science, considerable progress toward solving this challenge has been made using deep learning, in which multi-layer neural networks with millions of parameters are trained to generate peptide sequences from observed spectra. The first such deep learning method, DeepNovo^2^, has been followed by at least 22 additional publications (reviewed in^3^).

The standard method for evaluating these *de novo* sequencing methods is to use a gold standard produced via database search. In this approach, mass spectrometry data derived from a single species is searched against the reference proteome for that species, yielding a ranked list of peptide-spectrum matches (PSMs). Including in the peptide database a collection of reversed or shuffled “decoy” peptides provides a rigorous way to set a threshold in this list of PSMs while controlling the false discovery rate (FDR) among the PSMs above the threshold^4^. The resulting set of high-confidence PSMs can be used either to train or evaluate a *de novo* sequencing model.

Some version of the above protocol has been used to develop labeled training and validation data for essentially every published deep learning *de novo* sequencing method. One exception is methods that use spectra from synthesized peptide sequences for training^5–7^. However, even in these cases, a gold standard derived from database search is used for evaluation of the method.

Unfortunately, creating a high quality gold standard set of labeled spectra can be tricky. One challenge is ensuring that the search strategy employs appropriate parameters. For instance, one widely used benchmark dataset^2^ used a search strategy that failed to account for missassigned isotopic peaks during the acquisition stage. This error led to frequently assigning a deamidation modification, when the observed mass shift was better explained by an isotopic mass shift on the precursor *m/z*^3^. A second challenge relates to the notion of train/test leakage, in which information used to train the model leaks into the evaluation procedure. In the *de novo* setting, a common mistake is to randomly segregate a given set of labeled spectra into training and test sets, without regard to the associated peptides. As a result, spectra generated by the same peptide sequence may occur in both the training and test sets. Such duplicated peptides give an unfair advantage to the sequencing method, and the leakage will be even more useful to parameter-rich methods that are capable of memorizing many features of the training data.

In this work, we revisit the nine-species benchmark dataset that was employed in the first deep learning *de novo* sequencing method, DeepNovo^2^. This is a widely used dataset, which has been employed for training or evaluation in at least 15 subsequent studies^6,8–21^. The setup is quite straightforward. The authors downloaded nine publicly available datasets, all of which were generated on a Thermo Scientific Q Exactive mass spectrometer, and each of which was carried out in a different species. Each dataset was searched against the corresponding reference proteome, using a target-decoy strategy to accept a set of PSMs subject to a PSM-level FDR threshold of 1%. Because the data are derived from different species, the peptides in each set are largely (but not entirely) disjoint. To use the benchmark, it is typical to apply a cross-validation strategy, in which a model is trained on eight species and tested on the held-out species, and the procedure is repeated nine different ways.

In developing our Casanovo *de novo* sequencing model, we identified several problems with the nine-species benchmark^21^. These included the deamidation problem mentioned above, as well as some uncertainty regarding how the FDR was controlled. Perhaps most importantly, we recognized that a non-negligible proportion of peptides are shared among the different species, with the highest overlap between human and mouse.

In light of these difficulties, we downloaded the same datasets from the PRIDE repository and systematically reanalyzed all of the data, using a standard search procedure—the Tide search engine^22^ followed by Percolator^23^ with PSM-level FDR control at 1%. We then filtered the PSMs to prevent any peptide sequence from appearing in more than one species. The resulting data set was used to evaluate Casanovo^21^. Finally, because some of the single-species datasets are markedly larger than others, we produced a more balanced version of the dataset. Hence, we make publicly available both versions of this dataset: the peptide-disjoint dataset that can be used to avoid train/test leakage (“main”), and the reduced peptide-disjoint dataset if you want your analysis to run more quickly (“balanced”). In addition, we make available all of the intermediate files, for use in validating the benchmark.

## Methods

### Data sets

For our benchmark, we used the same nine studies originally identified by Tran *et al*.^2^.

1. Paiva *et al*. investigated the protein expression response of the cowpea plant (*Vigna unguiculata*) to infection by *Cowpea severe mosaic virus* (CPSMV) by carrying out label-free proteomic analysis of cowpea leaves that were inoculated with CPSMV compared to mock inoculation controls^24^.
2. Nevo *et al*. studied a rare autosomal recessive lysosomal storage disorder, cystinosis, by carrying out SILAC proteomic analysis of engineered mouse cell lines that harbor a known pathogenic mutation of the causative gene, *CTNS*^25^.
3. Cassidy *et al*. evaluated two different analytical approaches for carrying out full proteome analysis while identifying short open reading frames: a high/low pH reversed phase LC-MS bottom-up approach and a semi-top-down strategy involving separation of proteins in a GelFree system followed by digestion and LC-MS analysis^26^. The experiments were carried out using the methane producing archaeon *Methanosarcina mazei*.
4. Reuss *et al*. carried out proteomic analyses on a series of minimized strains of the model bacterium, *Bacillus subtilis*, with genomes reduced by  ~ 36%^27^.
5. Petersen *et al*. performed proteomic analysis of *Candidatus endoloripes*, which are bacterial symbionts of the *Lucinidae* family of marine bivalves^28^.
6. Mata *et al*. characterized the proteome of the tomato pericarp at its ripe red stage^29^.
7. Seidel *et al*. analyzed the global proteomic stress response in wildtype and two yeast knockout strains for the gene PBP1^30^.
8. Hu *et al*. studied honeybees that exhibit a suite of behaviors (*Varroa* sensitive hygiene—VSH) associated with infection with the *Varroa destructor* virus^31^. Proteomic analysis was carried out on mushroom bodies and antennae of adult honeybees with and without VSH.
9. Cypryck *et al*. characterized extracellular vesicles released from human primary macrophages after infection with influenza A viruses^32^.

All nine studies were performed using a Thermo Scientific Q Exactive mass spectrometer.

We downloaded the RAW files from the corresponding PRIDE projects (Table 1) and converted them to MGF format using the ThermoRawFileParser v1.3.4^33^. We downloaded the corresponding nine UniProt reference proteomes and constructed a Tide index for each one, using Crux version 4.2. Note that, for one species (*Vigna mungo*) no reference proteome is available, so we used the proteome of the closely related species *Vigna radiata*.

**Table 1:** Two versions of the nine-species benchmark.

PRIDE	Species	UniProt	Files	Spectra	Main		Balanced		Pre	Frag
					PSM	Pep	PSM	Pep		
PXD005025^24^	*Vigna mungo*	UP000087766	24	932,848	108,402	11,638	102,255	11,557	20	0.05
PXD004948^25^	*Mus musculus*	UP000000589	13	306,786	25,522	5630	25,522	5630	10	0.05
PXD004325^26^	*Methanosarcina mazei*	UP000033058	72	3,728,183	267,183	15,220	100,485	11,934	10	0.05
PXD004565^27^	*Bacillus subtilis*	UP000001570	106	4,336,428	1,351,938	28,364	113,234	17,481	30	0.05
PXD004536^28^	*Candidatus endoloripes*	UP000094849	11	2,272,023	82,514	8080	82,514	8080	20	0.05
PXD004947^29^	*Solanum lycopersicum*	UP000004994	60	603,506	177,553	48,459	100,056	35,787	15	0.05
PXD003868^30^	*Saccharomyces-cerevisiae*	UP000002311	27	1,477,397	585,846	19,102	108,973	13,285	20	0.05
PXD004467^31^	*Apis mellifera*	UP000005203	17	823,169	194,604	21,081	102,285	18,630	20	0.05
PXD004424^32^	*H. sapiens*	UP000005640	26	684,821	44,555	10,848	44,555	10,848	20	0.02
Total			343	15,165,161	2,838,117	168,422	779,879	133,232		

Counts of the number of annotated spectra and distinct peptide sequences are provided for the main and balanced versions of the benchmark. The final two columns specify the precursor window size (in ppm) and fragment bin size (in Da) used in the database search step.

### Database search and FDR control

We assigned peptide labels to spectra using the Tide search engine followed by post-processing with Percolator. In creating the Tide index, we specified Cys carbamidomethylation as a static modification and allowed for the following variable modifications: Met oxidation, Asn deamidation, Gln deamidation, N-term acetylation, N-term carbamylation, N-term NH_3_ loss, and the combination of N-term carbamylation and NH_3_ loss by using the tide-index options --mods-spec 1M+15.994915, 1N+0.984016, 1Q+0.984016 --nterm-peptide-mods-spec 1X+42.010565, 1X+43.005814, 1X-17.026549, 1X+25.980265 --max-mods 3. Note that one of the nine experiments (*Mus musculus*) was performed using SILAC labeling, but we searched without SILAC modifications and hence include in the benchmark only PSMs from unlabeled peptides. Tide automatically added to each index a shuffled decoy peptide corresponding to each target peptide. Thereafter, each MGF file was searched against the corresponding index using the precursor window size and fragment bin tolerance specified in the original study (Table 1). The search engine employed XCorr scoring with Tailor calibration^34^, and we allowed for 1 isotope error in the selection of candidate peptides. All search results were then analyzed jointly per species using the Crux implementation of Percolator, with default parameters. For the benchmark, we retained all PSMs with Percolator q value  < 0.01. We identified 13 MGF files with fewer than 100 accepted PSMs, and we eliminated all of these PSMs from the benchmark. At this point in the processing pipeline, the dataset contains 2,898,611 annotated spectra (PSMs) drawn from 343 RAW files and associated with 168,422 distinct peptides.

### Avoiding train/test leakage

To avoid train/test leakage, we post-processed the PSMs to eliminate peptides that are shared between species. Among the 168,422 distinct peptides, we identified 4121 (2.4%) that occur in more than one species. For each such peptide, we selected one of the associated species at random and then eliminated all PSMs containing that peptide in other species. Note that when identifying shared peptides between species, we considered all modified forms of a given peptide sequence to be the same, and we converted all isoleucines to leucines. Hence, if a given peptide appears in more than one species, then that peptide, including all its modified forms, is randomly assigned to a single species and eliminated from the others. The final, non-redundant benchmark dataset (“main”) consists of 2,838,117 PSMs corresponding to 168,422 distinct peptides.

### Balancing the benchmark

At this stage, the benchmark was quite imbalanced, in the sense that some species had a much larger number of associated PSMs. We therefore used a random downsampling procedure to produce a benchmark that is more evenly balanced across species. Among the nine species, the one with the fewest PSMs is *Mus musculus*, with 25,522. Downsampling all of the other eight species to have 25,000 PSMs would reduce the size of the dataset from 2.8 million PSMs to 225,000—a reduction of 92%. To avoid producing such a small dataset, we therefore opted to downsample each dataset to approximately 100,000 PSMs. This approach yields a slight imbalance, because three species have fewer than 100,000 PSMs (44,555 for *H. sapiens* and 82,514 for *Candidatus endoloripes*), while retaining a larger percentage of the original data. Our downsampling procedure involved randomly permuting the order of the MGF files for each species and then selecting the files in order until at least 100,000 PSMs have been accepted. The final, balanced benchmark dataset is approximately one quarter the size of the main benchmark, consisting of 779,879 PSMs from 133,232 distinct peptides.

## Data Records

The dataset^35^ contains files resulting from various steps in the generation of the benchmark:

- Spectrum files in MGF format, produced by ThermoRawFileParser.
- Reference proteome files in FASTA format, downloaded from UniProt.
- Search results files for both targets and decoys, in tab-delimited format, produced by Tide.
- PSM-level Percolator results files for targets, in tab-delimited format.
- Annotated MGF and corresponding mzSpecLib^36^ files for both versions of the benchmark (main and balanced).

Also included are log files for the steps of the analysis pipeline carried out using Crux^37^ (Tide indexing, Tide search, and Percolator). The data is available at 10.5281/zenodo.13685813.

## Technical Validation

**Fig. 1:**
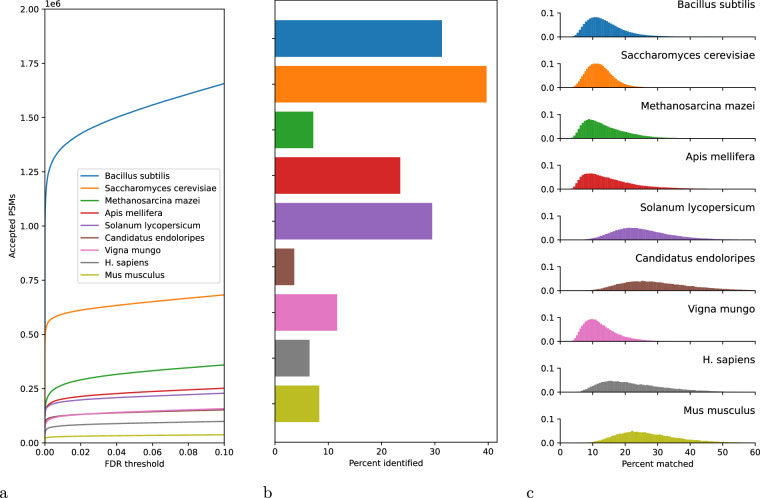
Validation of the benchmarks. (**a**) Each series indicates, for a given species, the number of accepted PSMs as a function of PSM-level FDR. (**b**) The bar plot indicates the proportion of spectra that were accepted at 1% PSM-level FDR per species. (**c**) Each histogram shows, for one species, the distribution of the proportion of total ion current that is matched by b- or y-ions per accepted PSM, using a matching tolerance of 0.05 *m/z*.

Data quality and interpretability varies dramatically from study to study, due to differences in sample type, sample preparation protocols, chromatography and instrument settings, and database size. To assess the overall rate of successful identification of spectra in each data set, we plotted the number of accepted PSMs as a function of PSM-level FDR threshold (Fig. 1a). As is typical in proteomics database search, the curves go up rapidly before leaving the y-axis, corresponding to the many spectra with highly confident peptide assignments. To better understand the relative quality of the datasets, we also computed the proportion of spectra that were accepted at 1% PSM-level FDR per species (Fig. 1b). Here we observe that some datasets yield much higher rates of accepted PSMs than others, up to 39.7% for *Saccharomyces cerevisiae* and down to 3.6% for *Candidatus endoloripes*. Despite this large variance in the rate of accepted PSMs, characterizing the proportion of the total peak intensities that is explained by matched b- and y-ions (Fig. 1c) suggests that the quality of the accepted PSMs is high. Notably, the proportion of matched b- and y-ions does not appear to be strongly correlated with the rate of accepted PSMs per species.

## Data Availability

All code required to generate the various benchmarks and to produce the figures in this manuscript is available with an Apache license at https://github.com/Noble-Lab/multi-species-benchmark, with a snapshot of the repository stored at 10.5281/zenodo.12926326.
